# The Belsey Mark IV: an operation with an enduring role in the management of complicated hiatal hernia

**DOI:** 10.1186/1471-2482-13-24

**Published:** 2013-07-05

**Authors:** Charalampos Markakis, Periklis Tomos, Eleftherios D Spartalis, Pavlos Lampropoulos, Leonidas Grigorakos, Dimitrios Dimitroulis, Elias Lachanas, E Andreas Agathos

**Affiliations:** 1 2nd Propaedeutic Department of Surgery, Department of Thoracic Surgery, Laiko General Hospital, University of Athens, Tritonos 20 Str. Paleo Faliro, 17561 Athens, Greece; 2 Department of Cardiothoracic Surgery, Athens Medical-Psychiko Clinic, Athens, Greece; 3 Department of Pulmonary Medicine, Neon-Athinaion, Athens, Greece

**Keywords:** Hiatal hernia, Belsey Mark IV, Thoracic approach

## Abstract

**Background:**

The Belsey Mark IV operation has been used for the management of hiatal hernia for over 40 years, but with the introduction of laparoscopic techniques its role has become questionable. To determine the current role of this procedure we present a contemporary series of patients.

**Methods:**

We reviewed fifteen consecutive patients, mean age of 63 years, who underwent a Belsey Mark IV fundoplication for gastroesophageal reflux in the presence of a hiatal hernia in our Department from January 2005 to March 2011. Indications for the thoracic approach included paraesophageal hernias, recurrent hiatal hernias and previous upper abdominal surgery.

**Results:**

There was no operative mortality. Immediate postoperative morbidity included 1 case of bleeding, 1 case of pneumonia and 1 case of atrial fibrillation. The mean length of stay was 5.9 days. After a mean follow-up time of 49 months, all patients reported total or partial alleviation of their symptoms. No hernia recurrence was detected during barium swallow examination.

**Conclusions:**

The Belsey approach is a procedure that can be useful as an alternative in selected cases when there are co-morbidities complicating the transabdominal (laparoscopic) approach.

## Background

The classic 1961 paper by Skinner and Belsey resulted in the widespread adoption of a surgical technique they named Belsey Mark IV (BMIV), the development of which had begun 20 years earlier. Based upon advances in the understanding of the anatomy and physiology of the gastroesophageal junction achieved in the 1950s and perfected after multiple clinical trials, the procedure’s results were finally published after it had been used on over 1000 patients, with a success rate of 85% [1,2]. The operation had been a mainstay in the management of hiatal hernia/gastrointestinal reflux disease (GERD) for over 40 years. However, the introduction of laparoscopic techniques in the 1990s resulted in the operation falling into disfavor in recent years.

Our group has used BMIV as the primary treatment of hiatal hernia/GERD until laparoscopic techniques became widely available. However, in the past 6 years a small number of patients has been referred to us and operated via a thoracic approach. In this report we present a series of consecutive patients operated via the BMIV in an effort to not only provide a reminder of a useful technique, but also identify possible indications for its continuing use in an era where hiatal hernia surgery is predominated by laparoscopic techniques.

## Methods

### Patients

The charts and outcomes of 15 consecutive patients with hiatal hernias treated using a BMIV fundoplication in our Department between January 2005 and March 2011 were reviewed. The ethical review board of the University of Athens approved our study and permitted us to collect and analyze patient data. All patients agreed to participate in the study and informed consent was obtained from each patient, to publish their treatment details including intraoperative photographs. There were 11 men and 4 women with a mean age of 63 years (38–79 years). All patients reported heartburn, 4 patients reported regurgitation, while no patients experienced preoperative dysphagia. In addition, 5 patients complained of atypical GERD symptoms such as coughing, chest and abdominal pain, and bloating. Two of the patients with large paraesophageal hernias reported recurrent aspiration. Indications for surgery via a thoracic approach were GERD symptoms refractory to medical therapy and/or endoscopic findings of esophagitis in 4 patients with previous abdominal surgery and/or marked obesity, large paraesophageal hernias in 4 patients, a gastroesophageal junction over 5 cm above the hiatus irreducible in barium swallow in 2 patients and hernia recurrence after previous surgery in 5 patients. Preoperatively all patients underwent esophagogastroscopy, which revealed signs of oesophagitis in 11 out of 15, barium swallow examination, and a computed tomographic scan. 24 hour pH monitoring, was performed in patients where no paraesophageal hernia or obvious signs of moderate to severe oesophagitis were present and was abnormal in 7 out of 8 patients.

### Surgical technique

**Figure 1:**
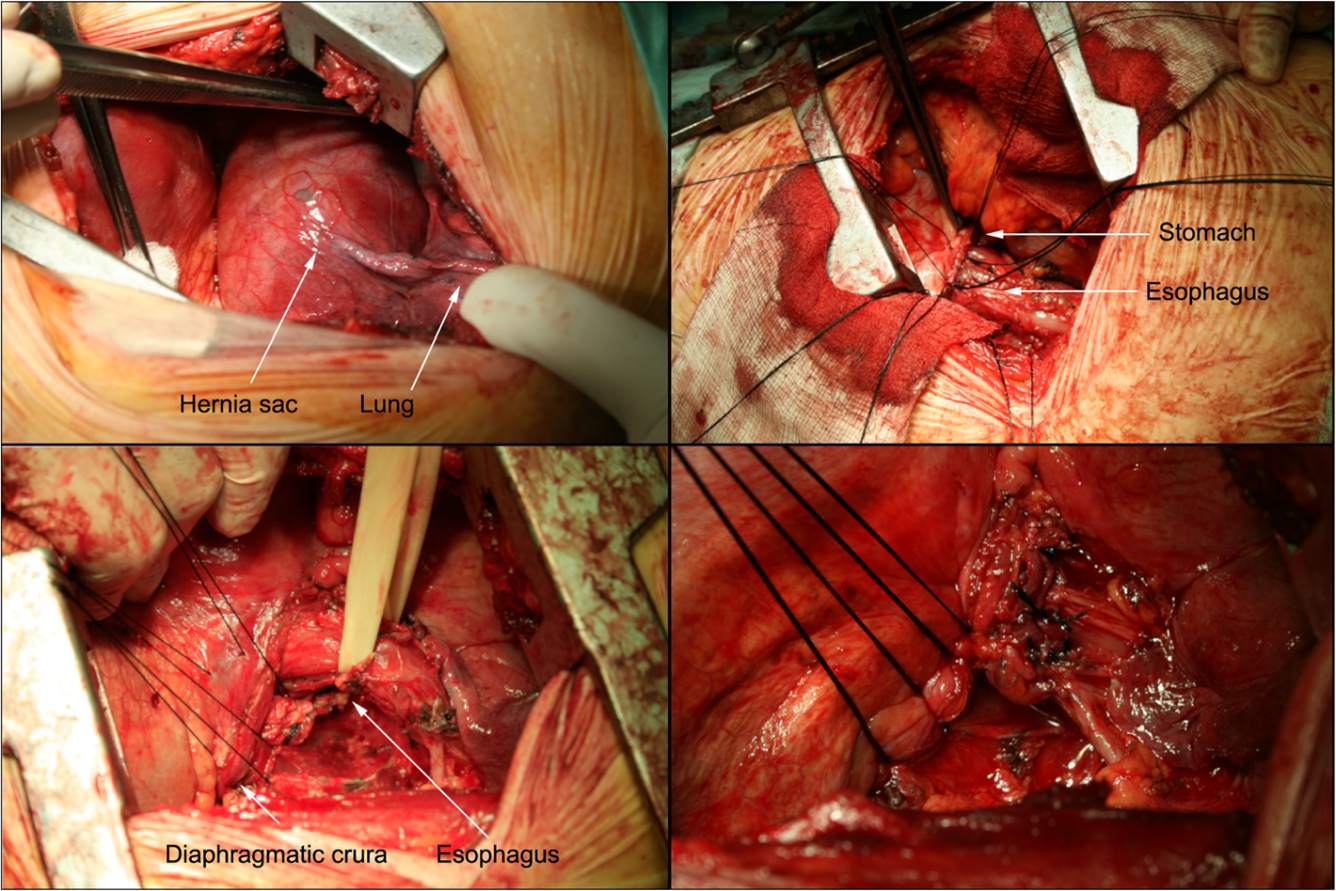
**Belsey Mark IV operation. Top left** – Hernial sac adhering to the lung. **Bottom left** – Sutures placed between the diaphragmatic crura. **Top right** – First row of sutures between the stomach and the esophagus. **Bottom right** – Second row of sutures incorporating the stomach, the esophagus and the diaphragm.

All patients had a double-lumen endotracheal tube. Before induction of anesthesia an epidural catheter was placed to facilitate postoperative pain control. The surgical approach was via a left lateral thoracotomy through the 6th or 7th intercostal space, with the patient in a right lateral decubitus position. Dissection and incision of the mediastinal pleura were performed as needed up to the level of the aortic arch. The hernial sac was dissected off the diaphragm. The esophagus was elevated using a penrose drain. Cephalad traction was placed on the esophagus and the phrenoesophageal membrane was incised circumferentially. The fundus of the stomach was mobilized, the fat pad excised, while the vagus nerves were preserved. The diaphragmatic crura (or more commonly the right and left bundles of the right crus) were then approximated posteriorly by 3–4 interrupted 0 silk sutures, which were left untied. An evaluation of the adequacy of the esophageal mobilization was then made and, if necessary, further mobilization was performed. The fundus was pulled up, 3 horizontal mattress sutures were placed 1.5-2 cm from the esophagogastric junction between stomach and esophagus to create the 270° wrap and these were then tied. Afterwards, the second row of sutures was placed 1–1.5 cm proximally so as to include the diaphragm and, after reduction of the fundus into the abdomen, these were tied also (Figure 1). Finally, the sutures between the crura were tied up to the point where a finger could pass easily through the hiatus. A pleural drainage tube was then placed and the thoracotomy closed. Analgesia was maintained with epidural bupivacaine, nonsteroidal anti-inflammatory drugs and systemic opiates, as needed. The patients were examined with an upper gastrointestinal series on the 4th postoperative day and they discharged from the hospital on the 5th or 6th postoperative day.

### Outcome assessment

Patients’ symptoms were evaluated before surgery, at 3, 6, 12 months after surgery and annually thereafter. At the time of this study on September 2011, all patients were interviewed. They were questioned about the presence, intensity (mild, moderate, severe) and frequency (daily, weekly, monthly, less frequently) of heartburn, dysphagia, regurgitation, pulmonary symptoms, nausea, vomiting, abdominal bloating, chest pain and the need for acid-reducing medications. Patients were specifically asked to describe post thoracotomy pain on a 4-point scale (1 pain free, 2 slight pain, negligible, 3 moderate pain requiring pain relief, 4 severe pain, intolerable), The Visick score, which consists of a 4- grade scale, was used to score the overall effect of surgery. All patients had esophagogastroscopy. When persistence or recurrence of GERD symptoms was noted, they were further examined with a barium swallow examination and 24 hour pH monitoring.

## Results

### Patient outcomes

The mean operative time was 144 minutes and the mean number of days until discharge 6. There was no mortality in our group of patients. Postoperative complications in 3 (20%) patients included one case of significant bleeding, one case of pneumonia and one case of atrial fibrillation. There were no esophageal or gastric perforations or other major morbidity. The mean follow-up time was 49 months (5–82). During the last follow-up (September 2011), 10 patients reported that they were completely free of symptoms; while 5 patients were symptomatic, 3 of which were still taking acid-reducing medication. Esophagogastroscopy showed that mucosal damage had subsided in 13 patients (Table 1). Almost half of the patients complained of some pain or discomfort at the thoracotomy site. 5 patients were symptomatic. Barium swallow examination and pH monitoring were ordered for all 5 symptomatic patients. No hernia recurrence was observed. pH monitoring was abnormal in 3 out of 5 patients. However, 1 patient with normal pH monitoring was symptomatic and reported alleviation of symptoms with medication (Table 2).

**Table 1:** Patient outcome by indication

**Indication for a thoracic approach**	**Esophagitis**		**Visick score**		**Post-thoracotomy pain**		Successful** surgery
	**Before surgery**	**After surgery**					
**Previous abdominal surgery**	2/4	0/4	I	2/4	1	3/4	4/4
			II	2/4	2	1/4	
					3	0/4	
GEJ* >5 cm above the hiatus	2/2	0/2	I	2/2	1	2/2	2/2
			II	0/2	2	0/2	
					3	0/2	
**Recurrence**	3/5	2/5	I	2/5	1	1/5	2/5
			II	5/5	2	2/5	
					3	2/5	
**Paraesophageal hernia**	4/4	0/4	I	4/4	1	2/4	4/4
			II	0/4	2	2/4	
					3	0/4	
**Total**	11/15	2/15	I	10/15	1	8/15	12/15
			II	5/15	2	5/15	
			III	0/15	3	2/15	
			IV	0/15	4	0/15	

*GEJ, gastroesophageal junction; **see Results in text.

**Table 2:** Overview of symptomatic patients

**Patient**		**Indication**	**Heartburn**	**Regurgitation**	**Dysphagia**	**Esophagitis**	**24-Hr pH**	**Medication**
**1**	Pre	Recurrence	Moderate	Moderate	Absent	SM II	Abnormal	PPi
	Post		Moderate		Mild	Absent	SM I	Abnormal	PPi
**2**	Pre	Recurrence	Mild	Absent	Absent	SM I	Abnormal	PPi	
	Post		Mild	Absent	Absent	SM I	Abnormal	PPi	
**3**	Pre	Recurrence	Mild	Absent	Absent	SM I	Abnormal	PPi	
	Post		Mild	Mild	Absent	No esophagitis	Normal	PPi	
**4**	Pre	Previous abdominal surgery	Moderate	Absent	Absent	SM I	Normal	PPi	
	Post		Mild	Absent	Absent	No esophagitis	Abnormal	None	
**5**	Pre	Previous abdominal surgery	Moderate	Absent	Absent	SM II	Abnormal	PPi	
	Post		Absent	Absent	Mild	No esophagitis	Normal	None	

*SM*, Savory-miller classification; *Pre*, Preoperatively; *Post*, Postoperatively; *PPi*, proton pump inhibitors.

Surgery was deemed as successful (absence of significant reflux symptoms without medication and no signs of esophagitis) in 80% (12/15) of patients. Two patients continued to experience symptoms and had mild esophagitis on endoscopy. The patients were evaluated by barium swallow examination and pH monitoring. The patients’ symptoms were satisfactorily controlled with proton pump inhibitors and no further intervention was proposed. A third patient reported continued symptoms which responded to medication (PPIs). The patient had normal pH monitoring and no mucosal damage in endoscopy and did not receive any other treatment.

## Discussion

The guidelines for the surgical treatment of gastroesophageal reflux disease issued by the Society of American Gastrointestinal and Endoscopic Surgeons in November 2010 do not mention the transthoracic approach as an option for the treatment of either primary or recurrent GERD [3]. Even though these guidelines do not address the management of paraesophageal hernia it is still interesting how an operation widely performed with excellent results for more than four decades [4] fell into disfavour so quickly after the transition to laparoscopic techniques. Thoracic surgeons traditionally involved in antireflux surgery, have joined in this rapid transition, having embraced the new laparoscopic techniques early on [5]. In the past decade a number of studies have suggested the use of a Belsey-Mark IV operation in selected indications or groups of patients.

Although BMIV is seldom performed as a primary antireflux operation in noncomplicated cases, many authors have proposed it as part of a tailored approach. Coosemans et al. proposed it in patients with esophageal dysmotility (although they acknowledged the alternative of a partial laparoscopic fundoplication), in case of extreme obesity, large hiatal hernias, previous abdominal surgery, redo surgery and in cases where the cardia appears to be irreducible on barium swallow. They correlated the last indication to esophageal shortening due to fibrosis and insisted on a thoracic route as essential in order to perform maximal esophageal mobilization and assess the necessity of a lengthening procedure [6]. Alexiou et al. reserved the use of BMIV for cases with presence of impaired oesophageal contractility or abnormal wave progression and reported a successful outcome in 83.1% of patients [7]. Kauer et al. used preoperative evaluation to choose patients with irreducible hernia or motility disorders for a thoracic approach. Patients with poor contractility or wave progressions were treated with a Belsey and those with a short esophagus with a Collis-Belsey procedure. In a group of 85 patients, 31 of which had a complicated disease, they reported an overall 89% success rate [8].

Patients with morbid obesity and GERD have often shown improvement of symptoms following Roux-en-Y gastric bypass surgery. There are, however, cases of intractable GERD in this patient population and a Belsey operation has been proposed, although somewhat controversially, as an alternative [9,10].

Massive paraesophageal hernias have been a classic indication for the transthoracic approach but recently the laparoscopic approach has been advocated and shown to be feasible, with good results [11]. However, the rarity of these cases and the difficulty in the technique of the laparoscopic approach, combined with the frequent presence of esophageal shortening are reasons cited to consider the Belsey Mark operation as an option [12]. There is no consensus as to the best approach for this subgroup of patients [11], despite a number of publications on the subject. It is important to note, however, that the transthoracic approach has been used in a primary repair and a redo repair group in a study that showed that it can be used safely for redo operations [13].

Other pathologies in the left side of the chest which can be simultaneously treated are an additional indication [4]. In a series of 62 patients operated between 1997 and 2001, 11 had a Belsey Mark fundoplication as a primary operation for GERD/hiatal hernia and indications included hiatal hernias fixed in the chest, esophageal diverticula, diffuse esophageal spasm and an oesophago-gastric junction tumor [12]. In this series, dysmotility disorders were treated with laparoscopic partial fundopliction. The same authors also reported combined operation for lung cancer, left-sided pneumothorax and bolus emphysema.

Redo surgery after an anti-reflux operation carries significant morbidity and results are good in about 70% of patients. The trend in reoperative surgery is for an abdominal and, if possible, laparoscopic approach. However, a recent review has reported use of the thoracic approach in 22% of cases [14]. In a report of 130 re-operations, the BMIV procedure was performed for patients with a migrated intrathoracic wrap with satisfactory results, although with a somewhat higher morbidity [15]. The authors propose the thoracic approach as a conversion strategy if the operation has been started laparoscopicaly but the intramediastinal dissection is difficult and the wrap cannot be freed. In another series reporting on 126 reoperations, BMIV was used as the procedure of choice for 25% of patients [16]. The authors again emphasized that the current trend toward laparoscopic repair should be interpreted with caution, since the fairly good results reported may be difficult to reproduce outside dedicated centers [3,16]. It must be emphasized that a short esophagus cannot be accurately predicted preoperatively [16] and any surgeon specialized and interested in esophageal reoperative surgery should be able to utilize a transthoracic approach with morbidity comparable to that of the open approach [17].

BMIV by video-assisted thoracic approach has been advocated to address two of the most serious drawbacks of the open transthoracic approach: poor cosmetic results and post-thoracotomy pain. However, the less than satisfactory results and higher complication rates reported with this technique have hindered its adoption [6,18,19]. The continued evolution of video-assisted thoracic surgery might renew interest in the transthoracic approach in the future.

## Conclusions

Our experience with the BMIV procedure involves a heterogeneous group of patients, referred to us by several general surgeons, which makes it difficult to speculate on the size of this subgroup relative to the total number of patients operated by the laparoscopic approach. Although we chose the thoracic approach on a case by case basis after the referring general surgeons expressed reservations about the risk posed by, or the appropriateness of, the abdominal route, the absence of a standardized referral system does not ensure that our patients had consulted an expert laparoscopic surgeon. It is therefore difficult to ensure that even relative contraindications for an abdominal/laparoscopic approach existed in our group of patients, except in two cases, where we were called to the operating room after laparoscopy revealed dense adhesions in the upper abdomen of previously operated patients.

We acknowledge that experienced laparoscopic surgeons can safely and efficiently treat the majority of cases of hiatal hernia by the minimally invasive transabdominal route. However, the BMIV procedure is still valuable and can provide an essential alternative to the laparoscopic approach, especially for the most complex cases such as reoperative surgery, where it is used on 20% of patients, and large paraesophageal hernias. Since the number of patients likely to be managed via a thoracic approach is limited and the Belsey operation is perhaps one of the technically more challenging, adequately training junior surgeons is also a major issue. Consequently, this operation should be used by surgeons experienced and interested in esophageal surgery, after careful consideration of alternative techniques.

Our patient group had satisfactory results overall (80%) considering the complexity of the case mix. However, 3 cases were considered as failures. Patients with failed surgery should be analyzed and managed carefully. To avoid failure a systematic approach to the preoperative workup is essential in order to avoid misdiagnosis (i.e. achalasia). Meticulous surgical technique can prevent an overly tight or, more commonly, overly loose wrap. However, continued reflux can be a problem in almost 15% of patients [4]. Re-operation should be offered with caution especially when no hernia recurrence can be detected, as was the case in our patients.

In conclusion, we have tried to describe the subgroup of patients with hiatal hernia where a transthoracic approach should be considered, keeping in mind that there are no conditions where the BMIV is routinely indicated. However, we believe that the BMIV procedure can still play a role in the modern management of GERD. This is an operation which is well worth preserving 50 years after its inception.

## Abbreviations

BMIV: Belsey Mark IV; GERD: Gastrointestinal reflux disease.

## Competing interests

The authors declare that they have no competing interest.

## Authors’ contributions

CM, EL and PT participated in the design of the study. CM, ES and PL wrote the article. AA and LG drafted and edited the manuscript. CM, PT and DD performed the revisions to the manuscript. All authors read and approved the final manuscript.

## Pre-publication history

The pre-publication history for this paper can be accessed here:

http://www.biomedcentral.com/1471-2482/13/24/prepub
